# Beyond deficits: which executive functions link to which NSSI behaviors? A network analysis

**DOI:** 10.3389/fpsyg.2026.1857988

**Published:** 2026-06-24

**Authors:** Yang He, Songyue Hong, Yajun Tian, Lin He, Keiwei Sun, Tao Xu

**Affiliations:** 1 School of Psychology, Shanghai Normal University, Shanghai, China; 2 Psychology Section, Secondary Sanatorium of Air Force Healthcare Center for Special Services, Hangzhou, China; 3 Department of Military Medical Psychology, Air Force Military Medical University, Xi’an, China

**Keywords:** adolescents, executive function, inhibitory control, network analysis, non-suicidal self-injury

## Abstract

**Background:**

Executive function deficits have been demonstrated to be closely associated with non-suicidal self-injury (NSSI) among adolescents. However, no studies to date have explored how the dimensions of executive function relate to specific NSSI behaviors in a joint framework. This study aimed to examine the relationships between the two constructs at a fine-grained level and identify the central nodes and bridge nodes of their relationships using network analysis.

**Methods:**

In this cross-sectional study, 1,300 Chinese adolescents completed the Adolescent Executive Function Scale to assess self-reported inhibitory control, cognitive flexibility, and working memory, and the 12-item behavioral subscale of the Adolescent Non-Suicidal Self-Injury Assessment Questionnaire to measure specific NSSI behaviors. A regularized partial correlation network was estimated to investigate the connections between variables, and the expected influence (EI) and bridge expected influence (BEI) were calculated for each node.

**Results:**

In addition to connections within each community, network analysis revealed specific cross-community associations between executive function dimensions and NSSI behaviors. Inhibitory control was most strongly connected to B4 “Intentionally punching walls, tables, windows or the ground”; working memory was most strongly connected to B6 “Deliberately biting oneself”; and cognitive flexibility showed associations with B1 “Deliberately pinching oneself.” The most important central node was B2 “Deliberately scratching oneself,” and the critical bridge nodes were B4 “Intentionally punching walls, tables, windows or the ground” and IC “Inhibitory control.”

**Conclusion:**

These preliminary findings suggest that different dimensions of self-reported executive function difficulties exhibit distinct patterns of co-occurrence with specific NSSI behaviors. The central node and bridge nodes identified here may represent potential targets for future prevention and intervention research.

## 1 Background

Non-suicidal self-injury (NSSI) is defined as the deliberate, direct, and repeated destruction of one’s own body tissue without suicidal intent, in a manner not socially sanctioned (American Psychiatric Association, 2013; Nock, 2010). This behavior typically emerges during adolescence, with peak onset between the ages of 10 and 19 (Brown and Plener, 2017). Common manifestations include cutting, scratching, hitting, and burning (Nock, 2010). A recent meta-analysis comprising 686,672 children and adolescents estimated the global lifetime prevalence of NSSI to be 22.1% (Lim et al., 2019), indicating that it is a widespread public health issue (De Luca et al., 2023). The prevalence among Chinese adolescents appears to be even higher, as a meta-analysis focusing on middle school students in mainland China reported an overall detection rate of 27.4% with an increasing trend year by year (Han et al., 2017). These high prevalence rates have serious consequences. NSSI is not only a clinical feature of various mental disorders such as depression and anxiety (Bentley et al., 2015), but also increases the risk of future suicidal behavior by approximately sevenfold (Dickstein et al., 2015). Notably, individuals who engage in NSSI often exhibit deficits in executive function, which may contribute to the onset and maintenance of self-injury (Fikke et al., 2011; Wang et al., 2023). Elucidating the specific links between these cognitive deficits and NSSI behaviors is therefore essential for understanding the mechanisms of NSSI.

Executive functions are higher-order cognitive processes that regulate thought, emotion, and behavior in the service of goal-directed action (Diamond, 2013), and are typically conceptualized as comprising three core dimensions: inhibitory control, working memory, and cognitive flexibility (Miyake et al., 2000). These functions can be measured through two distinct approaches: performance-based tasks administered in controlled settings, and self-report questionnaires that capture individuals’ everyday experiences of cognitive difficulty. The two methods assess different aspects of executive functioning and are typically only modestly correlated (Toplak et al., 2013). Self-report measures have been shown to predict meaningful real-world outcomes, including academic performance, health behaviors, adaptive function, and suicide-related risk (Pentz et al., 2015; Saffer and Klonsky, 2017; Daunhauer et al., 2017). The present study therefore focuses on self-reported executive difficulties as perceived in daily life, a construct that is related to but distinct from performance-based executive function. In parallel, performance-based and neuroimaging studies have consistently demonstrated that adolescents with NSSI exhibit poorer executive function compared to healthy controls (Plener et al., 2012; Wang et al., 2023). Executive dysfunction may render individuals more susceptible to becoming immersed in negative information and struggling to shift attention away from it (Grahek et al., 2019), while also impairing their capacity to inhibit self-injurious impulses (Roca et al., 2019). This dual impairment may help explain why some individuals, when confronted with negative emotions, have difficulty deploying adaptive regulation strategies and instead turn to self-injury as a maladaptive alternative.

Although the overall association between executive dysfunction and NSSI is well established, studies examining the specific dimensions of executive function in relation to NSSI have yielded inconsistent findings. For inhibitory control, although deficits are commonly reported (Allen and Hooley, 2015; Burke et al., 2021), some evidence indicates that individuals with more severe NSSI may exhibit enhanced reactive inhibition (Mirabella et al., 2024). Working memory deficits have been observed in adolescents with NSSI and their siblings (Fikke et al., 2011; Hu et al., 2021), yet a meta-analysis did not find a significant overall effect, suggesting a context-dependent role (Shen et al., 2022). Most studies have not found a direct link between cognitive flexibility and NSSI (Polanco-Roman et al., 2015; Shen et al., 2022). These inconsistencies may stem from methodological limitations of previous research. Most studies have assessed NSSI using aggregate frequency or total scores, which may overlook the heterogeneity of specific self-injurious behaviors that differ considerably in form, function, and physical consequences (Fried, 2015; Fried and Nesse, 2015; Bildik et al., 2013). Consequently, these approaches lack a fine-grained understanding of the specific pathways linking particular executive deficits to particular NSSI behaviors. Neglecting this heterogeneity may mask differential relationships and contribute to the inconsistent findings across studies (Fried et al., 2016; Fried and Cramer, 2017). Therefore, examining these relationships at a more detailed, variable-level is essential to advance mechanistic understanding and identify potential targets for precision intervention.

Network analysis, an emerging data-driven method, offers a powerful tool to address these limitations. This approach conceptualizes psychological variables as nodes and the statistical relationships between them as edges, visualizing the intrinsic structure of complex systems through graphical networks (Borsboom and Cramer, 2013; Epskamp et al., 2018a). From this perspective, psychopathological processes are understood as dynamic systems emerging from the interplay among symptoms and cognitive factors, rather than being attributable to a single underlying deficit (Borsboom, 2017). Compared to traditional approaches, network analysis can clarify fine-grained associations between specific executive function dimensions and NSSI behaviors, identify central nodes that are strongly connected to other nodes in the network, and pinpoint bridge nodes that connect different symptom communities (Robinaugh et al., 2016; Jones et al., 2021). Such nodes hold promise as targets for future intervention research (McNally, 2016). This method has been successfully applied to explore interrelationships among NSSI behaviors (Reinhardt et al., 2025), as well as comorbidity with other constructs (Xie et al., 2024). However, no study has employed network analysis to examine the links between executive function dimensions and NSSI behaviors.

To address this gap, the present study aims to construct a joint self-reported executive function–NSSI network, systematically investigating the patterns of interaction among the three core dimensions of self-reported executive function and 12 specific NSSI behaviors. We will compute centrality indices and bridge centrality indices to identify core nodes within the network and key bridge nodes connecting the two constructs. We hypothesize that dimensions of self-reported executive function will exhibit cross-community connections with specific NSSI behaviors, and that influential core and bridge nodes will be identified. This study aims to provide new theoretical insights into the mechanisms underlying adolescent NSSI at a fine-grained cognitive-behavioral level, and to identify potential targets for future prevention and intervention research.

## 2 Methods

### 2.1 Participants and ethical approval

This study was conducted via an online survey hosted on the Wenjuanxing platform.^1^ Using a convenience sampling method, participants were recruited during the fall semester of 2025 from senior high schools and vocational technical schools in Xinjiang, Shanxi, and Zhejiang provinces, China. Because the present study focuses on NSSI among senior high school and vocational school students, the target age range was set at 15–19 years.

The inclusion criteria were as follows: (1) being a currently enrolled senior high school or vocational technical school student; (2) being aged 15–19 years; (3) having no self-reported history of diagnosed neurological or psychiatric illnesses; and (4) voluntarily participating and providing informed consent from both the participants and their parents or legal guardians. A total of 1,500 questionnaires were initially obtained. The exclusion criteria were (1) failing to pass the attention check items (*n* = 78); (2) providing incorrect basic information, such as mismatched demographic data or logically inconsistent responses (*n* = 54); and (3) submitting incomplete questionnaires, defined as having more than 20% missing data on key study variables (*n* = 68). After applying these exclusion criteria, 200 invalid responses were removed, and the final sample comprised 1,300 adolescents.

The study protocol was approved by the Ethics Committee of Air Force Hangzhou Special Recreation Centre (No. TLZX20241129-01) and performed in accordance with the Declaration of Helsinki. All participants and their parents or legal guardians provided written informed consent prior to the survey, and anonymity and confidentiality were assured.

### 2.2 Measures

#### 2.2.1 Adolescent executive function scale

The adolescent executive function scale (AEFS) is a 21-item scale evaluating executive function (Huang et al., 2014). It includes three dimensions: inhibitory control, cognitive flexibility, and working memory, with items rated on a 3-point Likert scale ranging from 1 (never) to 3 (often). Higher scores indicate poorer executive function. In this study, Cronbach’s α coefficients were 0.84 for inhibitory control, 0.88 for cognitive flexibility, and 0.89 for working memory, and 0.83 for the total scale.

#### 2.2.2 Adolescent non-suicidal self-injury assessment questionnaire

The adolescent non-suicidal self-injury assessment questionnaire (ANSAQ) is a 31-item scale assessing non-suicidal self-injury (Wan et al., 2018), comprising a 12-item behavioral subscale and a 19-item functional subscale. In the current study, only the behavioral subscale was used, as our focus was on specific NSSI behaviors rather than underlying functions or motivations. The behavioral subscale measures the frequency of 12 specific self-injurious behaviors (e.g., “Intentionally cutting or scratching my skin with a sharp object,” “Banging my head against a hard object”) over the past year. A 5-point Likert scale is used, with each item containing five response options: “no” (0), “occasionally” (1), “sometimes” (2), “often” (3), and “always” (4). Higher total scores indicate more frequent NSSI behaviors. The scale demonstrated excellent internal consistency in this study, with a Cronbach’s α coefficient of 0.96.

### 2.3 Data analysis

Descriptive statistics (means and standard deviations) were calculated using SPSS 25.0 software. Network analysis was performed using R software (version 4.1.1).

#### 2.3.1 Network model of estimation and visualization

The R package qgraph was used to build and visualize the network of executive function and NSSI (Epskamp et al., 2012). The network was estimated via the Gaussian graphical model (GGM) (Epskamp et al., 2018b). In network building, the combined use of least absolute shrinkage and selection operator (LASSO) regularization and the extended Bayesian information criterion (EBIC) can attenuate trivial edges to zero, producing a sparse and interpretable network (Friedman et al., 2008; Foygel and Drton, 2010; Epskamp and Fried, 2018). Meanwhile, we set the EBIC hyperparameter to 0.5 to balance sensitivity and specificity in identifying true edges (Epskamp and Fried, 2018; Foygel and Drton, 2010).

In the model, nodes represented the three dimensions of the AEFS (inhibitory control, cognitive flexibility, and working memory) and the 12 behavioral items of the ANSAQ. Each edge represents the partial correlation between two nodes after statistically controlling for all other nodes in the network (Epskamp and Fried, 2018), with the estimation based on nonparametric Spearman correlations (Epskamp et al., 2018b). In the network visualization, blue and red edges represent positive and negative correlations, respectively, with edge thickness and color saturation reflecting the magnitude of the correlation.

#### 2.3.2 Central and bridging symptoms

Network centrality was calculated to quantify the extent to which each node is connected to other nodes in the network. Expected influence (EI), defined as the sum of all edge weights extending from a given node to all other nodes, was selected as the centrality index because it accounts for both positive and negative edges (Robinaugh et al., 2016). Higher EI values indicate that a node is more central or more influential within the network. EI values were computed using the R package qgraph (Epskamp et al., 2012).

To identify bridging nodes between the executive function and NSSI communities, bridge expected influence (BEI) was calculated. BEI is defined as the sum of the edge weights connecting a given node to all nodes in the other community (Jones et al., 2021). A higher BEI value indicates a greater likelihood that a node may activate symptoms in the other community. In the present network, nodes were divided into two communities: the executive function community (three nodes) and the NSSI community (12 nodes). BEI values were calculated using the R package networktools (Jones et al., 2021).

#### 2.3.3 Network accuracy and stability

The accuracy and stability of the network were evaluated using the R package *bootnet* (Epskamp et al., 2018a). First, the accuracy of edge weights was examined with bootstrapped 95% confidence intervals (CIs) based on 1,000 bootstrap samples. Narrower CIs indicate more accurate estimation of edge weights (Mullarkey et al., 2019). Second, the correlation stability (CS) coefficient, calculated by a case-dropping bootstrap approach (1,000 bootstrap samples), was used to evaluate the stability of the EI and BEI estimations. A CS coefficient greater than 0.5 indicates strong stability, while a value greater than 0.25 is considered acceptable (Epskamp et al., 2018a). Third, bootstrapped difference tests (1,000 bootstrap samples) were conducted to compare edge weights, EIs, and BEIs.

## 3 Results

### 3.1 Sample characteristics

A total of 1,300 participants were included in the analysis. The demographic characteristics of the sample are summarized in Table 1. The mean age of the participants was 16.75 years (SD = 1.03). Table 2 presents the means, standard deviations, variable abbreviations, and raw EI and BEI values for each executive function dimension and NSSI item.

**Table 1:** Demographic characteristics of the adolescent participants (*N* = 1, 300).

Variables	*n* (%)/*M* (SD)
Age	16.75 (1.03)
Gender	
Male	499 (38.38)
Female	801 (61.62)
Only child	
Married	336 (25.89)
Unmarried/divorced/widowed	964 (74.19)
Family monthly income per capita (RMB)	
<3,000	250 (19.23)
3,000 ~ 5,000	655 (50.38)
>5,000	395 (30.38)
Place of residence	
Rural area	706 (54.32)
Urban area	594 (45.68)

**Table 2:** Abbreviations, mean scores, standard deviations, EIs (raw values), and BEIs (raw values) for each variable in the executive function-NSSI network.

Variables	Abb	*M*	SD	EI	BEI
Dimensions of executive function					
Inhibitory control	IC	8.69	2.43	0.84	0.13
Cognitive flexibility	CF	12.09	3.56	0.97	0.06
Working memory	WM	10.82	3.28	0.80	0.02
Symptoms of NSSI					
Deliberately pinching oneself	B1	1.19	0.55	0.96	0.07
Deliberately scratching oneself	B2	1.15	0.52	1.13	0.02
Intentionally banging one’s head against objects	B3	1.14	0.49	0.94	0.02
Intentionally punching walls, tables, windows or the ground	B4	1.23	0.60	0.81	0.13
Striking oneself with fists, slaps or hard objects	B5	1.14	0.49	0.99	−0.01
Deliberately biting oneself	B6	1.13	0.48	0.97	0.03
Pulling out one’s own hair intentionally	B7	1.12	0.47	0.82	0.03
Stabbing or piercing oneself deliberately	B8	1.09	0.43	1.07	−0.01
Deliberately cutting oneself	B9	1.09	0.43	0.90	0.00
Burning or scalding oneself intentionally	B10	1.08	0.38	1.05	−0.01
Rubbing the skin with objects to cause bleeding or bruising	B11	1.09	0.42	1.01	0.00
Carving words or symbols into the skin	B12	1.08	0.40	0.79	0.00

Abb, abbreviation; M, mean; SD, standard deviation; EI, expected influence; BEI, bridge expected influence.

### 3.2 Network structure

**Figure 1:**
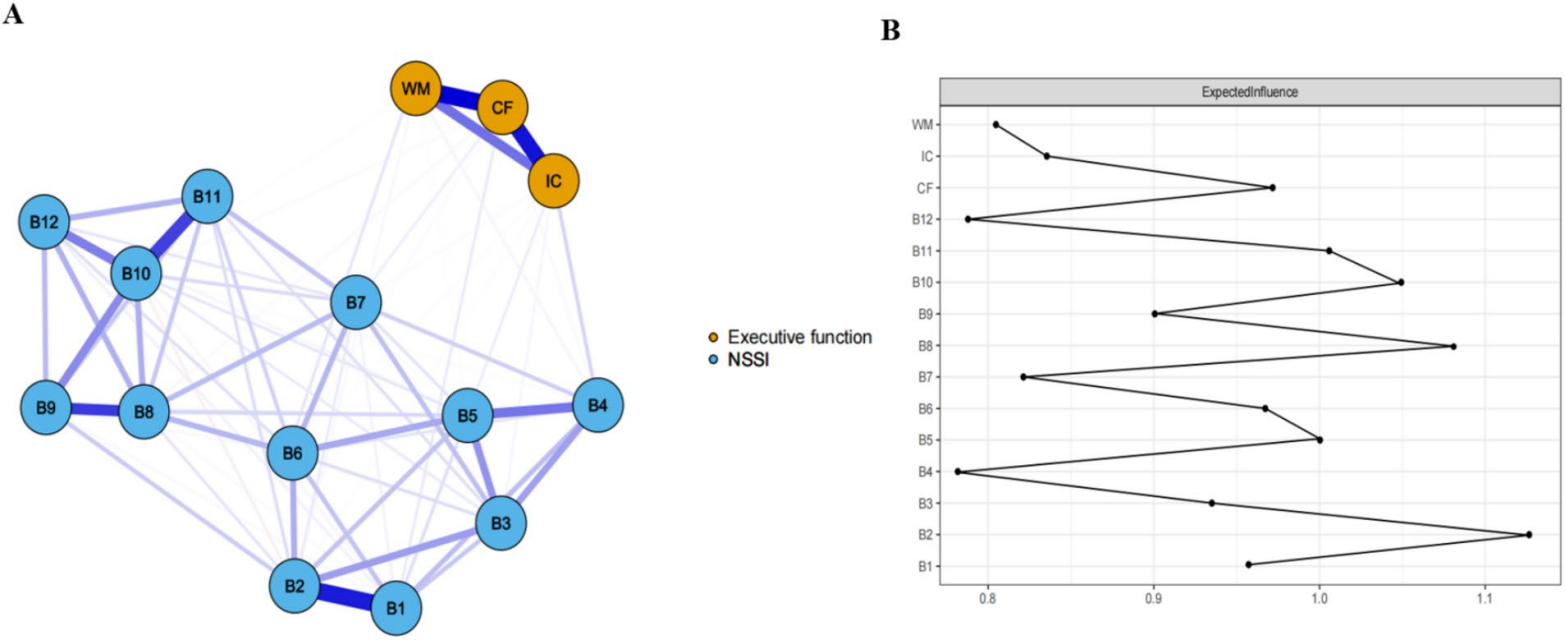
The executive function-NSSI network model in participants and EIs of the nodes in the network. **(A)** The executive function-NSSI network model in participants. **(B)** EIs of the nodes in the network (raw scores). The blue and red lines represent positive and negative correlations between executive function components and NSSI symptoms, and the thickness of the line and the saturation of the color represent the magnitude of the correlation. CF = cognitive flexibility; IC = inhibitory control; WM = working memory; B1 = deliberately pinching oneself; B2 = deliberately scratching oneself; B3 = intentionally banging one’s head against objects; B4 = intentionally punching walls, tables, windows or the ground; B5 = striking oneself with fists, slaps or hard objects; B6 = deliberately biting oneself; B7 = pulling out one’s own hair intentionally; B8 = stabbing or piercing oneself deliberately; B9 = deliberately cutting oneself; B10 = burning or scalding oneself intentionally; B11 = rubbing the skin with objects to cause bleeding or bruising; B12 = carving words or symbols into the skin.

The network model is shown in Figure 1A. This network exhibited several important characteristics. First, among the three strongest edges in the network, two were between executive function components: the edge between CF “Cognitive flexibility” and WM “Working memory” (weight = 0.47), and between IC “Inhibitory control” and CF “Cognitive flexibility” (weight = 0.44); the other one was between NSSI components: the edge between B1 “Deliberately pinching oneself” and B2 “Deliberately scratching oneself” (weight = 0.46). Second, there were several cross-community connections between executive function and NSSI; the strongest such edges were IC “Inhibitory control”–B4 “Intentionally punching walls, tables, windows or the ground” (weight = 0.07), WM “Working memory”–B6 “Deliberately biting oneself” (weight = 0.05), and CF “Cognitive flexibility”–B1 “Deliberately pinching oneself” (weight = 0.04). Third, most edges were positive; however, a few negative edges were observed, including those between B1 “Deliberately pinching oneself” and B10 “Burning or scalding oneself intentionally” (weight = −0.02), B2 “Deliberately scratching oneself” and B10 “Burning or scalding oneself intentionally” (weight = −0.02), B4 “Intentionally punching walls, tables, windows or the ground” and B10 “Burning or scalding oneself intentionally” (weight = −0.01), as well as between CF “Cognitive flexibility” and B8 “Stabbing or piercing oneself deliberately” (weight = −0.01), and between CF “Cognitive flexibility” and B10 “Burning or scalding oneself intentionally” (weight = −0.01). All edge weights within the executive function–NSSI network can be found in Supplementary Table S1, and the bootstrapped 95% confidence intervals indicate that the accuracy of the edge weights was relatively reliable (see Supplementary Figure S1). Supplementary Figure S2 presents the bootstrapped difference test for the edge weights.

### 3.3 Central symptoms

Node EI values were calculated to assess the relative importance of each node in the executive function-NSSI network (see Figure 1B; Table 1). Among all nodes, B2 “Deliberately scratching oneself” exhibited the highest EI value (EI = 1.13), establishing it as the central symptom in the network. The CS coefficient for EI was 0.75, indicating that the estimation of node EI possessed strong stability (see Supplementary Figure S3). The results of the bootstrapped difference test for node EI are presented in Supplementary Figure S4.

### 3.4 Bridge symptoms

**Figure 2:**
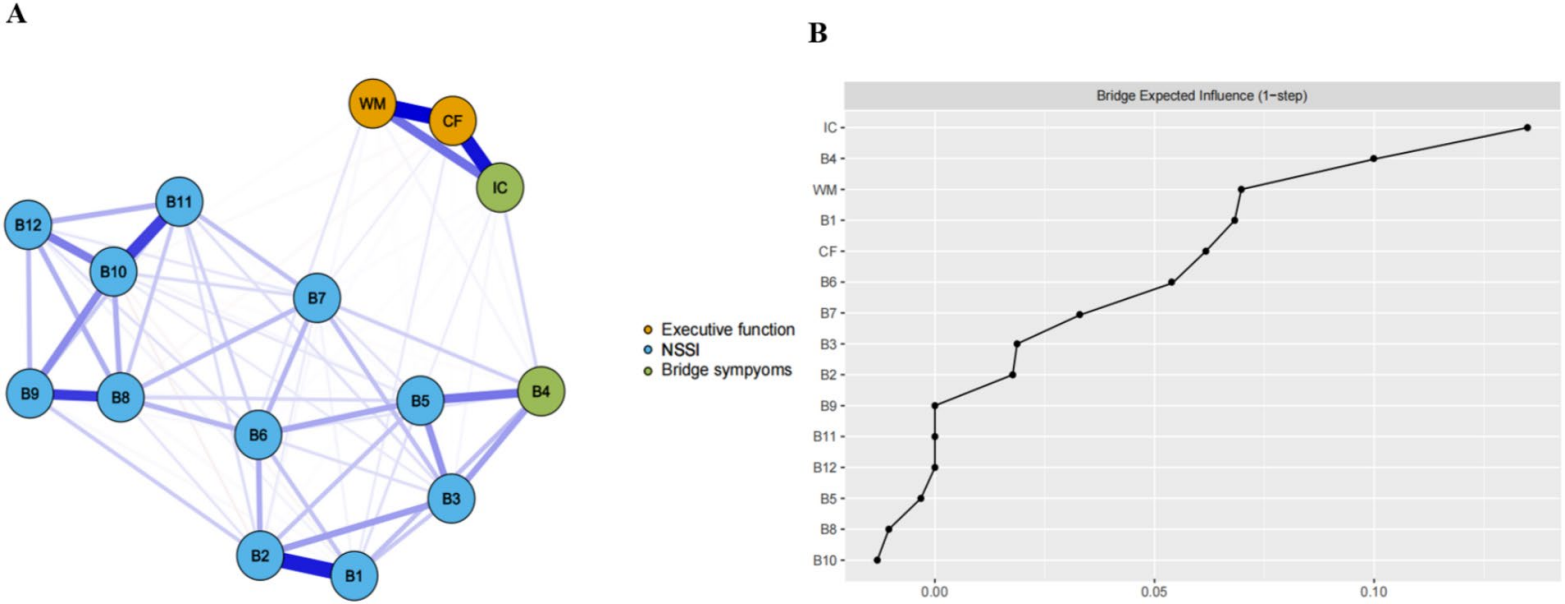
The bridge symptoms in the executive function-NSSI network of participants and BEIs of the nodes in the network. **(A)** The bridge symptoms in the executive function-NSSI network of participants. **(B)** The BEIs of nodes in the network (raw scores). The blue and red lines represent positive and negative correlations between executive function components and NSSI symptoms, and the thickness of the line and the saturation of the color represent the magnitude of the correlation. CF = cognitive flexibility; IC = inhibitory control; WM = working memory; B1 = deliberately pinching oneself; B2 = deliberately scratching oneself; B3 = intentionally banging one’s head against objects; B4 = intentionally punching walls, tables, windows or the ground; B5 = striking oneself with fists, slaps or hard objects; B6 = deliberately biting oneself; B7 = pulling out one’s own hair intentionally; B8 = stabbing or piercing oneself deliberately; B9 = deliberately cutting oneself; B10 = burning or scalding oneself intentionally; B11 = rubbing the skin with objects to cause bleeding or bruising; B12 = carving words or symbols into the skin.

As shown in Figure 2A, the BEI values for each node are displayed in Figure 2B. IC “Inhibitory control” (BEI = 0.13) and B4 “Intentionally punching walls, tables, windows or the ground” (BEI = 0.10) had the highest BEIs of their communities and thus were identified as bridges in the network. As shown in Supplementary Figure S5, the BEIs of B4 “Intentionally punching walls, tables, windows or the ground” and IC “Inhibitory control” were significantly larger than those of most other nodes in the network (*p* < 0.05). The CS coefficient for BEI was 0.75, exceeding the recommended threshold of 0.5 and signifying that the BEI estimation was adequately stable (see Supplementary Figure S6).

## 4 Discussion

This study is the first to estimate the network structure of self-reported executive function and NSSI based on individual dimensions and behaviors, and to investigate the relationships between these constructs using network analysis. In addition to connections within each community, we found specific associations between particular dimensions of self-reported executive function and distinct NSSI behaviors. These results suggest that there may be fine-grained associations linking subjectively perceived executive difficulties with NSSI. This study also identified several variables that may be particularly relevant to the co-occurrence of self-reported executive difficulties and NSSI, including the central node B2 “Deliberately scratching oneself,” as well as the bridge nodes IC “Inhibitory control” and B4 “Intentionally punching walls, tables, windows or the ground.” These findings offer preliminary implications for clinical practice, suggesting potential targets for further investigation in the prevention and intervention of NSSI among adolescents.

First, the strongest edges appeared within each community rather than in connections between the self-reported executive function and NSSI communities. This pattern is consistent with numerous previous network analysis studies on psychopathological comorbidity, which have consistently found that the strongest associations exist between symptoms or dimensions from the same domain (Borsboom, 2017; Niu et al., 2024; Zhang et al., 2025). Within the self-reported executive function community, the two strongest edges were observed between cognitive flexibility and working memory, and between inhibitory control and cognitive flexibility. These findings may be expected, as the core dimensions of executive function are intrinsically interrelated (Diamond, 2013; Friedman and Miyake, 2017).

For the NSSI community, the strongest edge was observed between “Deliberately pinching oneself” (B1) and “Deliberately scratching oneself” (B2). This finding aligns with recent network analyses conducted in diverse populations. Among adolescents with depressive disorders, the strongest connection within the NSSI subnetwork was precisely between pinching and scratching (Zhang et al., 2025; Xie et al., 2024). Extending this to community adults, severe scratching and pinching not only cluster together but serve as central “hub” behaviors with the strongest connections to other forms of self-injury in the overall network (Reinhardt et al., 2025). The convergence of findings across clinical, community, and general adolescent samples suggests that the strong association between these two behaviors is not population-specific but rather reflects a fundamental pattern in how NSSI behaviors co-occur. A plausible explanation is that both are superficial self-injury methods requiring no tools, and thus may tend to be employed by the same individuals (Lloyd-Richardson et al., 2007; Klonsky, 2011). Together, these findings support the theoretical notion that symptoms or dimensions within the same construct tend to cluster together, forming coherent behavioral syndromes.

In addition to within-community edges, we found that some dimensions of self-reported executive function were associated with specific NSSI behaviors. It should be noted that the edge weights for these cross-community associations were relatively modest (ranging approximately from 0.04 to 0.07), indicating small but statistically reliable associations rather than strong psychopathological pathways. For example, inhibitory control was most strongly connected to “Intentionally punching walls, tables, windows or the ground” (B4). Inhibitory control, as captured by the self-report measure used in this study, reflects individuals’ perceived capacity to suppress inappropriate impulses and behavioral responses in everyday contexts (Toplak et al., 2013); B4 “Intentionally punching walls, tables, windows or the ground” represents a highly impulsive, immediate self-injury method that may often occur during episodes of emotional dysregulation (Kimbrel et al., 2018; Nock, 2010). A possible explanation for this specific association is that individuals who perceive themselves as having poorer inhibitory control may be more likely to struggle with suppressing impulsive urges when confronted with negative emotions (Burke et al., 2021), which could increase the likelihood of engaging in this specific form of NSSI as an immediate behavioral response (Allen and Hooley, 2015). Critically, B4 has been identified as a key maintenance factor for NSSI, as research has shown that wall/object punching produces maximal relief from negative affect, thereby potentially creating a negative reinforcement loop that strengthens future use (Kimbrel et al., 2018). Furthermore, impulsivity has been found to be indirectly associated with wall/object punching through anger, suggesting the possibility that negative emotions, particularly anger, may trigger impulsive self-injury when self-perceived inhibitory control is compromised (Patel et al., 2022). Although Patel et al.’s sample consisted of veterans, this mechanism may also be relevant to adolescents, as anger is a common emotional precursor to NSSI across populations (Nock, 2010). Thus, the combination of self-perceived poor inhibitory control and the impulsive nature of “Intentionally punching walls, tables, windows or the ground” (B4) may partly account for why individuals who report executive difficulties are predisposed to this specific form of NSSI. It is noteworthy that IC “Inhibitory control” and “Intentionally punching walls, tables, windows or the ground” (B4) also showed the highest BEI values in their respective communities, meaning that inhibitory control has the greatest estimated influence on the NSSI community, while B4 has the greatest estimated influence on the self-reported executive function community. Statistically, intervening to target these bridge nodes might help reduce the co-occurrence of self-reported executive difficulties and NSSI (Jones et al., 2021); however, this possibility requires empirical testing.

Another small but statistically reliable association was found between cognitive flexibility and “Deliberately pinching oneself” (B1). This finding is consistent with previous findings that subjectively reported cognitive flexibility deficits exist in individuals with NSSI (Wang et al., 2023; Wang et al., 2025). A behavioral study found that among adolescents with first-episode drug-naïve depression, those with NSSI exhibited significant cognitive flexibility deficits on performance-based tasks, and that higher NSSI frequency was associated with poorer performance (Wang et al., 2023). Another study using latent variable models revealed that self-reported cognitive flexibility was negatively associated with NSSI among adolescents (Wang et al., 2025). Cognitive flexibility, as captured by the self-report measure used in this study, reflects individuals’ perceived capacity to switch between different tasks, rules, or mental states in everyday contexts (Toplak et al., 2013). As Uddin comprehensively reviewed, cognitive flexibility enables individuals to adapt their thoughts and behaviors in response to changing environmental demands; conversely, deficits in cognitive flexibility may lead to rigid, perseverative patterns of responding (Uddin, 2021). When individuals perceive themselves as cognitively inflexible, this may manifest as an inability to shift coping strategies when facing stress, potentially leading to a reliance on familiar but maladaptive behavioral patterns. One possible account is that self-perceived cognitive inflexibility could limit the diversity of available emotion regulation strategies, increasing the likelihood that individuals will fall back on repetitive, familiar patterns of responding to distress (Grant and Chamberlain, 2023; Lloyd-Richardson et al., 2023). Notably, B1 has been characterized as a low visibility and easily implementable self-injury method that requires no preparation and can be performed discreetly in any setting (Reinhardt et al., 2025). In this context, such a readily accessible behavior may become the “default” coping response for individuals who perceive themselves as unable to flexibly regulate their emotions. Therefore, the observed association between these two self-reported variables is plausible, although the underlying mechanisms require further investigation.

A similarly modest association was observed between working memory and “Deliberately biting oneself” (B6). Previous findings on the relationship between working memory and NSSI have been mixed. Some studies have documented working memory deficits on performance-based tasks in individuals with NSSI (Chen et al., 2023; Fikke et al., 2011; Hu et al., 2021). For instance, a behavioral study using the Spatial Working Memory task found that adolescents with high-severity NSSI exhibited significant working memory impairments (Fikke et al., 2011). However, other studies have reported null findings. One cross-sectional study found that adolescents with NSSI did not exhibit significant working memory impairments compared to healthy controls after controlling for IQ, suggesting that general intellectual ability may confound the relationship (Mürner-Lavanchy et al., 2022). Moreover, a recent meta-analysis reported no significant overall association between performance-based working memory and NSSI, highlighting the heterogeneity in this literature (Shen et al., 2022). These mixed findings raise the possibility that the relationship between working memory and NSSI may not be uniform across all forms of self-injury, but rather might depend on specific self-injury methods. From a self-report perspective, individuals who perceive their working memory as poor may have fewer cognitive resources available for flexible emotion regulation (Schmeichel et al., 2008; Etkin et al., 2015), which could potentially lead them to resort to more primitive, readily accessible self-injury methods. “Deliberately biting oneself” (B6) may represent such a method as it requires no tools, can be performed instantly, and provides immediate sensory stimulation (Reinhardt et al., 2025). Nevertheless, the observed association is modest, and further research is needed to clarify the specific conditions under which self-reported working memory difficulties relate to biting behaviors.

In line with our hypothesis, the node with the highest expected influence was “Deliberately scratching oneself” (B2). This finding aligns with previous studies showing scratching as a central node among depressed adolescents in both the childhood maltreatment–NSSI network (Xie et al., 2024) and the rumination-NSSI network (Zhang et al., 2025). Importantly, the present study extends prior research by revealing the central position of “Deliberately scratching oneself” (B2) specifically within the self-reported executive function and NSSI network, a context not previously examined. The centrality of this node may be attributable to its inherent characteristics. “Deliberately scratching oneself” (B2) is a superficial self-injury behavior with high prevalence among both adolescents (Idig-Camuroglu and Gölge, 2018) and adults (Reinhardt et al., 2025). It offers extremely high immediate accessibility, requiring no tools and allowing spontaneous execution (Lloyd-Richardson et al., 2007; Nock, 2010). This behavior can rapidly release emotional pressure while producing visible but typically non-serious skin damage (Nock, 2010). Furthermore, scratching often co-occurs with other NSSI behaviors, particularly pinching (Xie et al., 2024; Zhang et al., 2026; Reinhardt et al., 2025). Its high accessibility and low threshold might explain its central network position, as it could be connected to other self-injury methods. Notably, from a clinical perspective, as central nodes may be important for the overall network structure (Borsboom, 2017; Robinaugh et al., 2016), interventions targeting “Deliberately scratching oneself” (B2), such as replacement strategies (e.g., squeezing stress balls or clay) that provide alternative sensory stimulation (Xie et al., 2024), might help disrupt the broader NSSI network and mitigate symptom severity. This possibility, while intriguing, is speculative, and future longitudinal research is needed to examine whether changes in “Deliberately scratching oneself” (B2) indeed influence the broader network (Fried et al., 2017).

This study offers initial insights into the fine-grained relationships within the self-reported executive function and NSSI network. Nevertheless, several limitations should be acknowledged. First, the results were based on self-reported scales. Although anonymity was assured, self-reports may lead to subjective biases and social approval effects (Paulhus and Vazire, 2007; Ren et al., 2021). In particular, executive function was assessed exclusively through a self-report measure, which captures subjectively perceived cognitive difficulties rather than objectively measured cognitive abilities. Therefore, all interpretations concerning executive function in this study are confined to this self-report level. Future studies would benefit from incorporating both self-report and performance-based measures of executive function to provide a more comprehensive assessment. Second, the cross-sectional nature of the design precludes causal inferences. The network associations reported here represent statistical relationships and should not be interpreted as indicating causal or mechanistic pathways. Future studies should examine causal relationships using longitudinal experimental designs. Third, although we identified central and bridge nodes as potential intervention targets (Beard et al., 2016; Jones et al., 2021), further longitudinal or experimental research is required to determine whether interventions targeting these nodes would be effective. The present design cannot establish that modifying these nodes will produce clinical change. Fourth, our sample included only adolescents aged 15–19 years from high schools and vocational schools; the exclusion of younger adolescents aged 10–14 may have introduced selection bias, as the peak onset of NSSI occurs across the full 10–19 age range. Therefore, the findings should be understood as reflecting NSSI among senior high school and vocational school students rather than adolescent NSSI more broadly, and caution is warranted when generalizing the findings to younger adolescent populations or clinical samples. Fifth, convenience sampling was used in this study, which may affect the representativeness of the sample and limit the generalizability of the findings. Replication studies employing probability sampling methods in more diverse populations are needed to confirm the robustness of our results. Sixth, important psychopathological covariates, including depressive symptoms, anxiety symptoms, emotion dysregulation, and impulsivity, were not assessed in this study. These variables are known to be associated with both self-reported executive dysfunction and NSSI, and their absence is a significant limitation. It remains uncertain whether the observed network patterns are specific to self-perceived executive function or reflect broader psychological distress or general psychopathology. Future research should incorporate these covariates to evaluate the unique contribution of self-reported executive function dimensions to NSSI. Seventh, the cross-community edge weights identified in the network were relatively modest in magnitude. Although they were statistically stable, their small size suggests that other unmeasured factors may play important roles in linking self-reported executive difficulties to NSSI, and these connections should not be characterized as strong psychopathological pathways. Finally, the network characteristics are specific to the scales we used. We assessed executive function using the Adolescent Executive Function Scale (Huang et al., 2014) and NSSI using the behavioral subscale of the Adolescent Non-Suicidal Self-Injury Assessment Questionnaire (Wan et al., 2018). Consequently, the present study did not capture all aspects of these constructs. Future studies should consider other facets (e.g., NSSI functions, neuropsychological tasks of executive function) and integrate them into a unified framework to provide a more comprehensive understanding of the self-reported executive function and NSSI network.

## 5 Conclusion

In summary, this study represents an initial step in investigating the network structure of self-reported executive function dimensions and specific NSSI behaviors in adolescents. Within the self-reported executive function and NSSI network, B2 “Deliberately scratching oneself” emerged as the most central node, while IC “Inhibitory control” and B4 “Intentionally punching walls, tables, windows or the ground” emerged as bridge nodes connecting the two systems. These findings offer preliminary insights into how subjectively perceived executive difficulties may relate to specific NSSI behaviors and suggest potential targets for further clinical investigation. It must be emphasized that these results pertain to self-reported cognitive difficulties and cannot be directly equated with neurocognitive deficits. Given the cross-sectional design and the modest magnitude of cross-community associations, these findings should be considered exploratory. Future research, particularly longitudinal and experimental studies, is needed to examine whether these observed associations reflect causal pathways and whether the proposed targets are effective in clinical practice.

## Data Availability

The original contributions presented in the study are included in the article/Supplementary material, further inquiries can be directed to the corresponding authors.

## Footnotes

1
https://www.wjx.cn
