# Supplementary material for: Beyond deficits: which executive functions link to which NSSI behaviors? A network analysis
Source: Front Psychol. 2026 Jun 24;17:1857988. doi: 10.3389/fpsyg.2026.1857988 (PMC13341517; doi:10.3389/fpsyg.2026.1857988)
Supplement: Supplementary file 1 [file Supplementary_file_1.DOCX]

Supplementary materials

Supplementary Table S1 All edges weights within the network

|  | IC | CF | WM | B1 | B2 | B3 | B4 | B5 | B6 | B7 | B8 | B9 | B10 | B11 | B12 |
| --- | --- | --- | --- | --- | --- | --- | --- | --- | --- | --- | --- | --- | --- | --- | --- |
| IC |  | 0.44 | 0.26 | 0.03 | 0.00 | 0.02 | 0.07 | 0.00 | 0.00 | 0.00 | 0.00 | 0.00 | 0.00 | 0.00 | 0.00 |
| CF | 0.44 |  | 0.47 | 0.04 | 0.00 | 0.00 | 0.01 | 0.00 | 0.01 | 0.03 | -0.01 | 0.00 | -0.01 | 0.00 | 0.00 |
| WM | 0.26 | 0.47 |  | 0.00 | 0.01 | 0.00 | 0.02 | 0.00 | 0.05 | 0.00 | 0.00 | 0.00 | 0.00 | 0.00 | 0.00 |
| B1 | 0.03 | 0.04 | 0.00 |  | 0.46 | 0.10 | 0.12 | 0.06 | 0.11 | 0.02 | 0.01 | 0.01 | -0.02 | 0.00 | 0.02 |
| B2 | 0.00 | 0.00 | 0.01 | 0.46 |  | 0.18 | 0.00 | 0.11 | 0.15 | 0.04 | 0.05 | 0.09 | -0.02 | 0.06 | 0.00 |
| B3 | 0.02 | 0.00 | 0.00 | 0.10 | 0.18 |  | 0.17 | 0.20 | 0.06 | 0.11 | 0.00 | 0.00 | 0.04 | 0.05 | 0.00 |
| B4 | 0.07 | 0.01 | 0.02 | 0.12 | 0.00 | 0.17 |  | 0.25 | 0.05 | 0.08 | 0.00 | 0.00 | -0.01 | 0.00 | 0.00 |
| B5 | 0.00 | 0.00 | 0.00 | 0.06 | 0.11 | 0.20 | 0.25 |  | 0.16 | 0.05 | 0.07 | 0.04 | 0.04 | 0.00 | 0.01 |
| B6 | 0.00 | 0.01 | 0.05 | 0.11 | 0.15 | 0.06 | 0.05 | 0.16 |  | 0.13 | 0.13 | 0.01 | 0.00 | 0.08 | 0.06 |
| B7 | 0.00 | 0.03 | 0.00 | 0.02 | 0.04 | 0.11 | 0.08 | 0.05 | 0.13 |  | 0.12 | 0.00 | 0.07 | 0.11 | 0.05 |
| B8 | 0.00 | -0.01 | 0.00 | 0.01 | 0.05 | 0.00 | 0.00 | 0.07 | 0.13 | 0.12 |  | 0.30 | 0.15 | 0.10 | 0.14 |
| B9 | 0.00 | 0.00 | 0.00 | 0.01 | 0.09 | 0.00 | 0.00 | 0.04 | 0.01 | 0.00 | 0.30 |  | 0.21 | 0.10 | 0.13 |
| B10 | 0.00 | -0.01 | 0.00 | -0.02 | -0.02 | 0.04 | -0.01 | 0.04 | 0.00 | 0.07 | 0.15 | 0.21 |  | 0.35 | 0.24 |
| B11 | 0.00 | 0.00 | 0.00 | 0.00 | 0.06 | 0.05 | 0.00 | 0.00 | 0.08 | 0.11 | 0.10 | 0.10 | 0.35 |  | 0.14 |
| B12 | 0.00 | 0.00 | 0.00 | 0.02 | 0.00 | 0.00 | 0.00 | 0.01 | 0.06 | 0.05 | 0.14 | 0.13 | 0.24 | 0.14 |  |

*Note*: CF = cognitive flexibility; IC = inhibitory control; WM = working memory; B1 = deliberately pinching oneself; B2 = deliberately scratching oneself; B3 = intentionally banging one’s head against objects; B4 = intentionally punching walls, tables, windows or the ground; B5 = striking oneself with fists, slaps or hard objects; B6 = deliberately biting oneself; B7 = pulling out one’s own hair intentionally; B8 = stabbing or piercing oneself deliberately; B9 = deliberately cutting oneself; B10 = burning or scalding oneself intentionally; B11 = rubbing the skin with objects to cause bleeding or bruising; B12 = carving words or symbols into the skin.


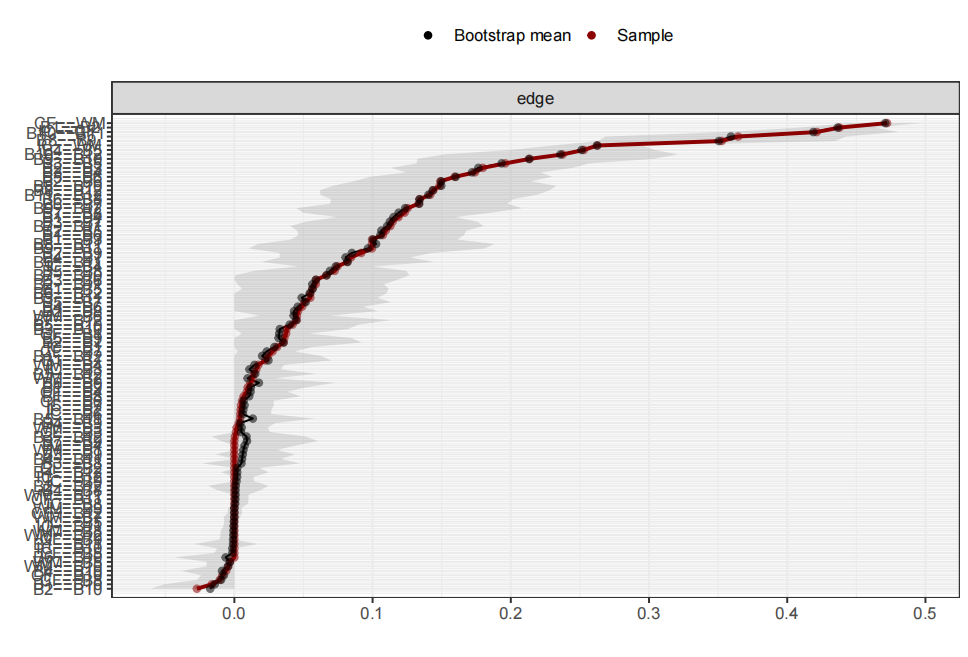


Supplementary Figure S1 Accuracy of edge weights in the network

*Note*: The red line depicts the sample edge weights and the gray bar depicts the bootstrapped confidence interval.


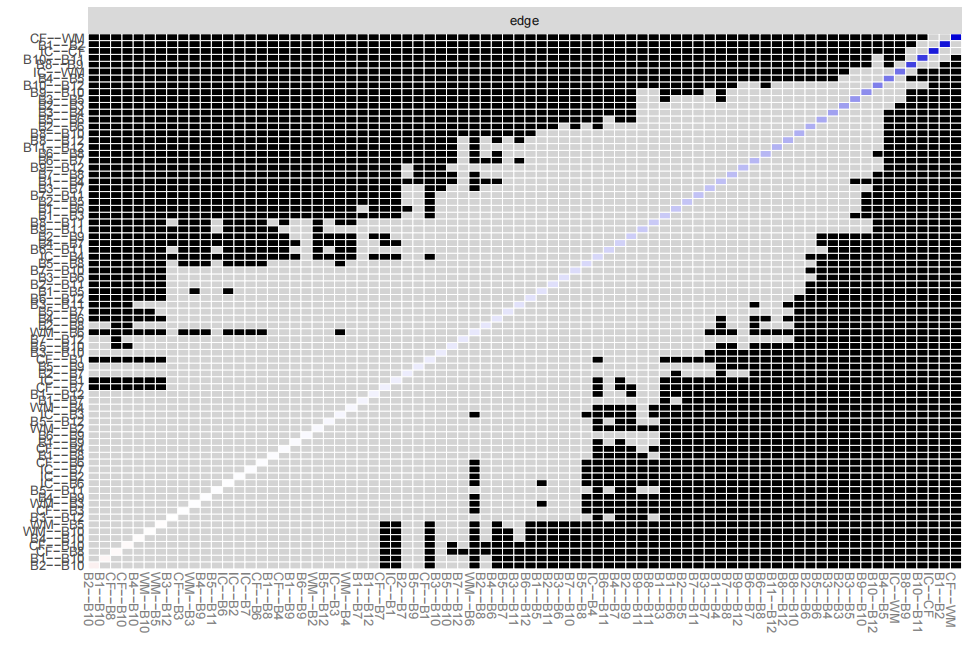


Supplementary Figure S2 Bootstrapped difference test for edge weights in the network

*Note*: Gray boxes indicate edge weights that do not differ significantly from one another, while black boxes indicate edge weights that do differ significantly. Blue and red boxes on the diagonal correspond to edge weights with positive and negative correlations, respectively.


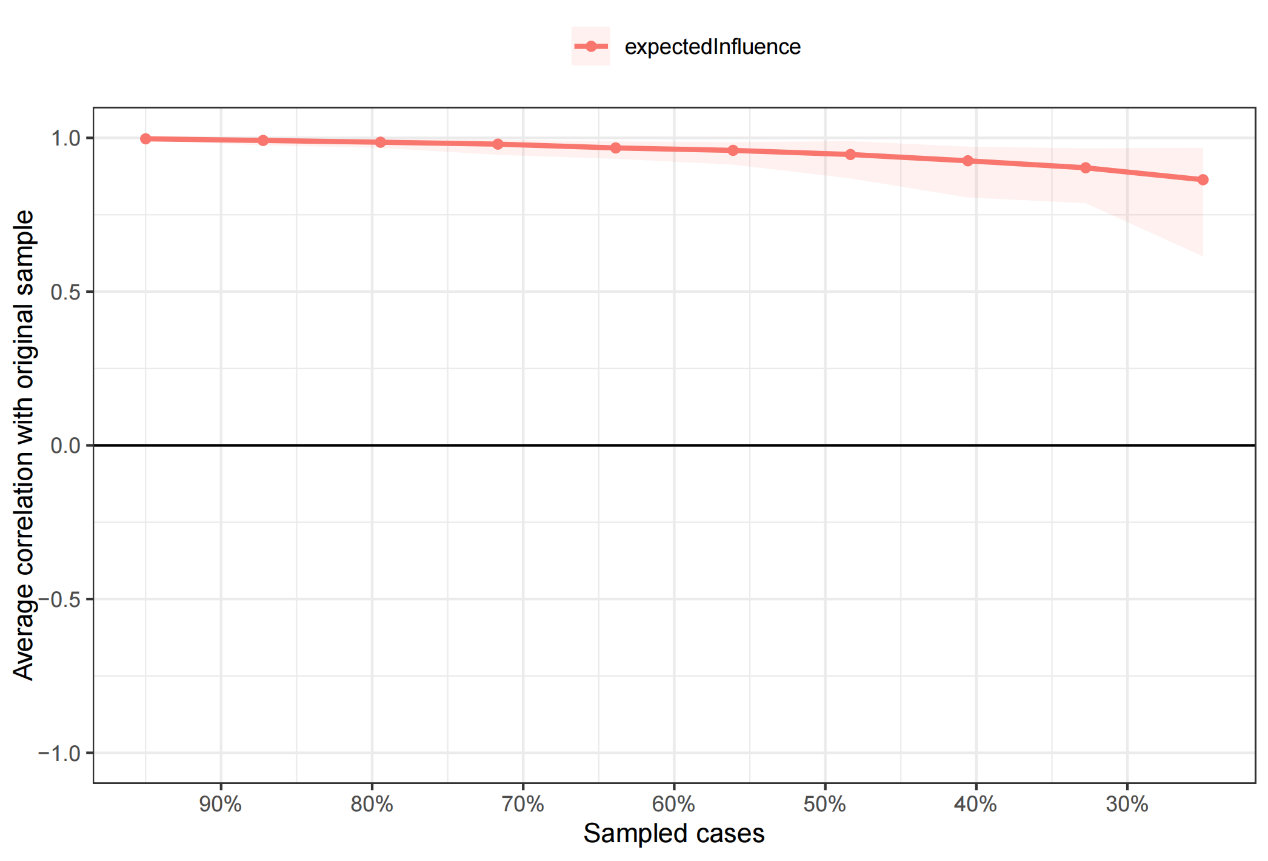


Supplementary Figure S3 Stability of node expected influences in the network

*Note*: The red bar represents the average correlation between node expected influences in the full sample and subsample with the red area depicting the 2.5th quantile to the 97.5th quantile.


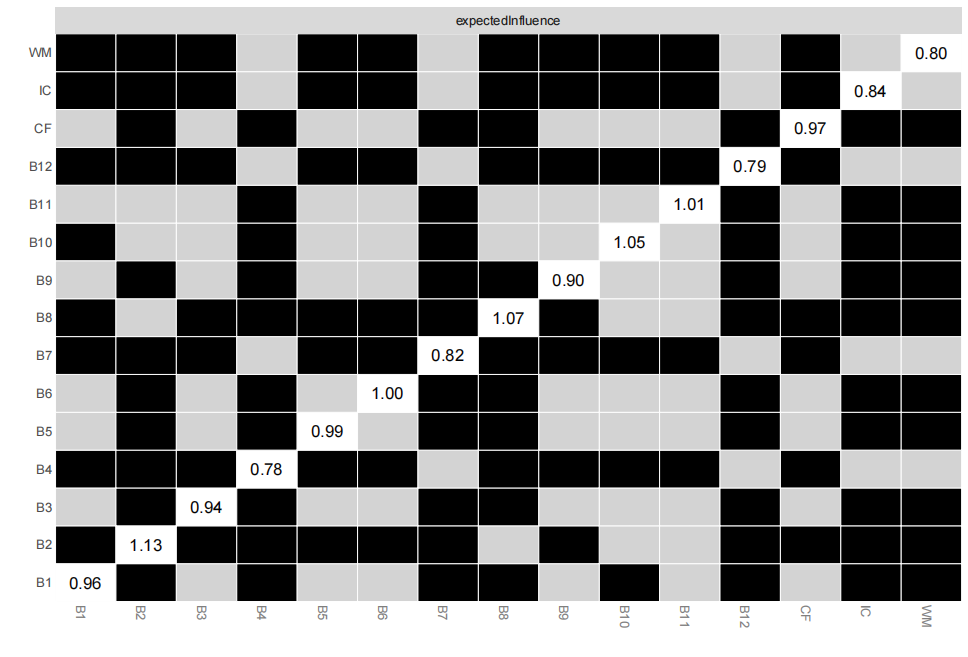


Supplementary Figure 4 Bootstrapped difference test for node expected influences in the network

*Note*: Gray boxes indicate node expected influences that do not differ significantly from one another, while black boxes indicate node expected influences that do differ significantly. The numbers in the white boxes (i.e., diagonal line) represent the values of node expected influences.


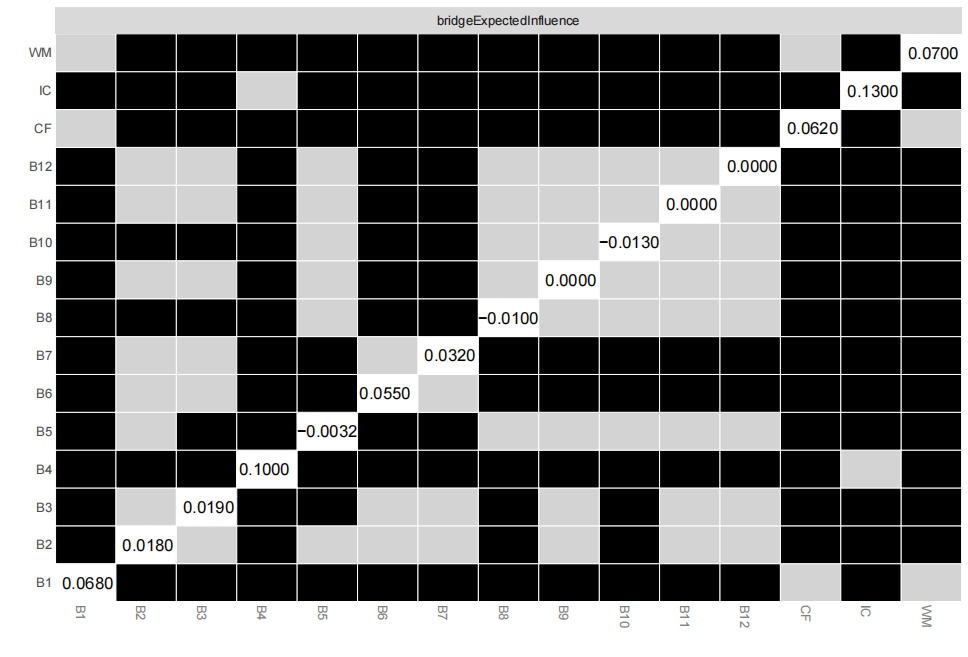


Supplementary Figure S5 Bootstrapped difference test for node bridge expected influences in the network

Note: Gray boxes indicate node bridge expected influences that do not differ significantly from one another, while black boxes indicate node bridge expected influences that do differ significantly. The numbers in the white boxes (i.e., diagonal line) represent the values of node bridge expected influences.


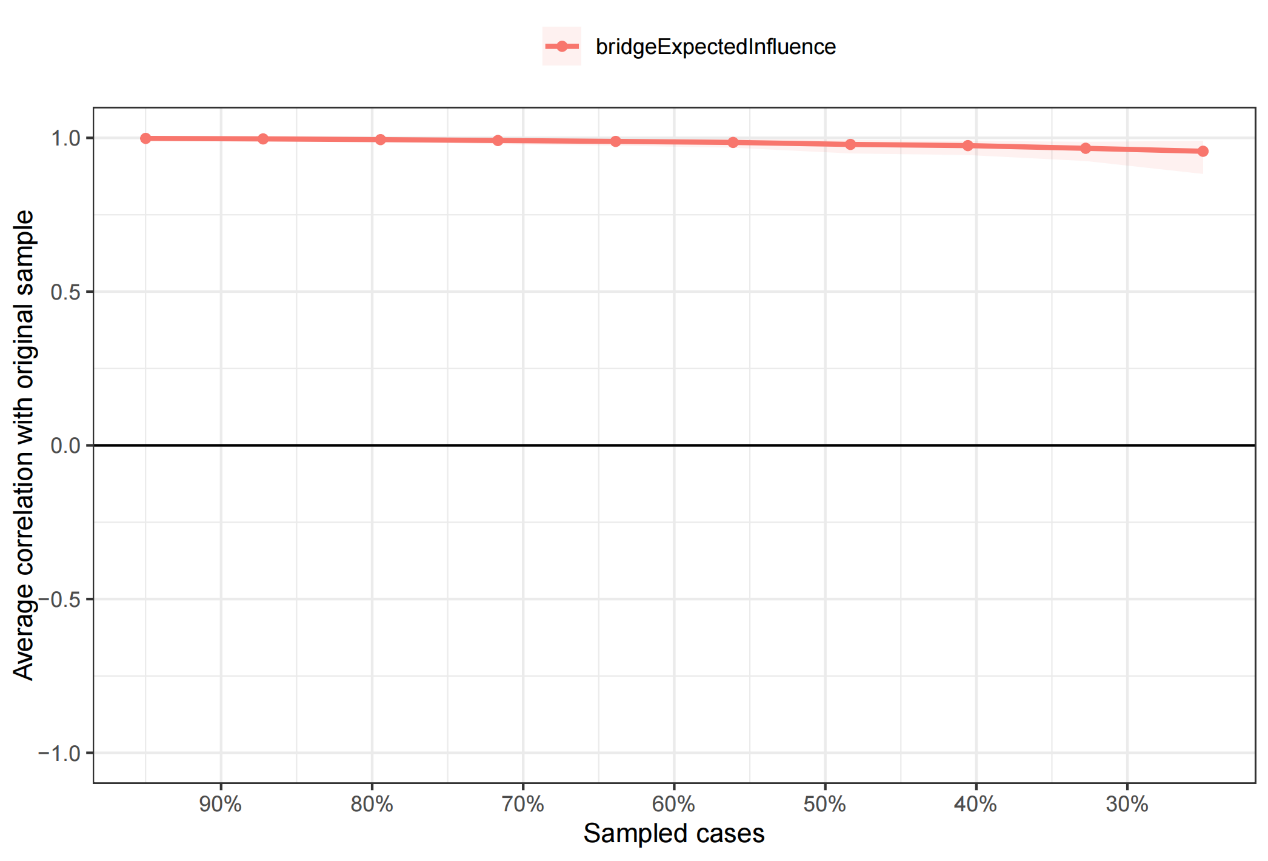


Supplementary Figure S6 Stability of node bridge expected influences in the network

Note: The red bar represents the average correlation between node bridge expected influences in the full sample and subsample with the red area depicting the 2.5th quantile to the 97.5th quantile.
